# The influence of ageing on the incidence and site of trauma femoral fractures: a cross-sectional analysis

**DOI:** 10.1186/s12891-019-2803-x

**Published:** 2019-09-05

**Authors:** Shao-Chun Wu, Cheng-Shyuan Rau, Spencer C. H. Kuo, Peng-Chen Chien, Ching-Hua Hsieh

**Affiliations:** 1 Department of Anesthesiology, Kaohsiung Chang Gung Memorial Hospital, Chang Gung University and College of Medicine, Kaohsiung, 83301 Taiwan; 2 Department of Neurosurgery, Kaohsiung Chang Gung Memorial Hospital, Chang Gung University and College of Medicine, Kaohsiung, 83301 Taiwan; 3 Department of Plastic Surgery, Kaohsiung Chang Gung Memorial Hospital, Chang Gung University and College of Medicine, No.123, Ta-Pei Road, Niao-Song District Kaohsiung City, 833 Taiwan

**Keywords:** Femur, Fracture, Trauma, Weight, Obese, Fall, Osteoporosis, Age

## Abstract

**Background:**

This study aimed to determine the influence of ageing on the incidence and site of femoral fractures in trauma patients, by taking the sex, body weight, and trauma mechanisms into account.

**Methods:**

This retrospective study reviewed data from adult trauma patients aged ≥20 years who were admitted into a Level I trauma center, between January 1, 2009 and December 31, 2016. According to the femoral fracture locations, 3859 adult patients with 4011 fracture sites were grouped into five subgroups: proximal type A (*n* = 1359), proximal type B (*n* = 1487), proximal type C (*n* = 59), femoral shaft (*n* = 640), and distal femur (*n* = 466) groups. A multivariate logistic regression analysis was applied to identify independent effects of the univariate predictive variables on the occurrence of fracture at a specific site. A two-dimensional plot was presented visually with age and the propensity score accounts for the risk of a fracture at a specific femoral site.

**Results:**

This analysis revealed that older age was an independent variable that could positively predict the occurrence of proximal type A (OR [95%CI]: 1.03 [1.03–1.04], *p* < 0.001) and B fractures (1.02 [1.01–1.02], *p* < 0.001), and negatively predict the occurrence of proximal type C (0.96 [0.94–0.98], *p* < 0.001), shaft (0.95 [0.95–0.96], *p* < 0.001), and distal fractures (0.98 [0.98–0.99], *p* < 0.001).

**Discussion:**

Using the propensity scores which account for the risk of a fracture in a specific femoral site, this study revealed that the older patients were at a higher risk of developing proximal type A and type B fractures, while a lower risk of developing fractures in the shaft and distal femur. This incidence of fracture site can largely be explained by age-related factors, including a decrease in bone strength and falling being the most common mechanism of trauma in older patients.

**Conclusions:**

This study revealed a difference in the involvement of age in the incidence of femoral fracture sites in the trauma patients.

## Introduction

Femoral fractures are an injury commonly seen in the emergency room. According to an 11-year long population-based cohort study in Taiwan, the adjusted standard incidence rate of hip fracture is between 5.01 and 11.70 per million persons. As the longest bone in the human body, the femur is divided into several different parts including the head, neck, greater and lesser trochanters, shaft, and the distal condyles. Fractures can occur in any of these areas. The fracture site is determined by the force, the impact point, and how the forces are transmitted through the bone [1]. In addition, the fracture site of the femur may also be determined by the structure and strength of the bone. The site of femoral fracture can be categorized according to the Arbeitsgemeinschaft für Osteosynthesefragen (AO) classification as proximal femoral (type A: trochanteric; type B: neck; and type C: head), femoral shaft, and distal femoral fractures [2].

Determination of the influence of age on the incidence of femoral fractures in any given part of the bone is complex, because many age-related factors, including gender, trauma mechanism, body weight, and bony density, would also have impact on the occurrence of the femoral fracture. The factors influencing femoral fracture site are interrelated and are not independent. For example, increasing age is associated with osteoporotic bone changes, which are believed to increase the rate of femoral fracture; falls occur more frequently in the elderly [3, 4] but traffic-related fractures caused by motorcycle or bicycle accidents occur more often in younger adults and the rate of its occurrence differs between genders [4, 5]. In a fall accident, the force directly impacts the posterolateral aspect of the greater trochanter, but the impact point is not limited to only this site in a non-fall accident. Therefore, proximal type A and B fractures are predominant in falls, but in motorcycle accidents, femoral shaft fracture comprises the most common fracture site, followed by distal femoral fractures [4]. In addition, The distribution of impact force through the hip that is associated with falls from a standing height is greater than the average energy required to fracture an elderly hip but not that of a younger adult [6]. Furthermore, gender is another important risk factor that has influence on the occurrence of femoral fracture, as a sharp decline in bone mineral density (BMD) among women during transition from 5th to 6th decade which signifies association of menopause with osteoporosis [7]. Obesity is another of the most important factor that determines possible osteoporotic change to a femur [8–10]. Previous studies have shown that the magnitude of traumatic impact force increases in proportion to body weight and thus would increase the incidence of fractures around the knee [11]. However, some studies report that obese patients have a lower rate of hip fracture [12–14], which may be due to increased cushioning of the bone by fat deposits over the trochanter and iliac wing areas [15, 16], and more importantly, increased bone density and less osteoporotic bone [17, 18].

Therefore, using the propensity scores which account for the risk of a fracture in a specific femoral site, this study aimed to determine the association of age with the site and incidence of femoral fracture in trauma patients, by taking the sex, body weight, and trauma mechanisms (fall and non-fall) into account.

## Methods

### Ethics statement

Ethical approval for this study was obtained from the institutional review board (IRB) of the Kaohsiung Chang Gung Memorial Hospital, a level I regional trauma center in southern Taiwan [5, 19, 20] (reference number: 201800742B0). According to IRB regulations, the need for informed consent was waived.

### Study population

**Fig. 1:**
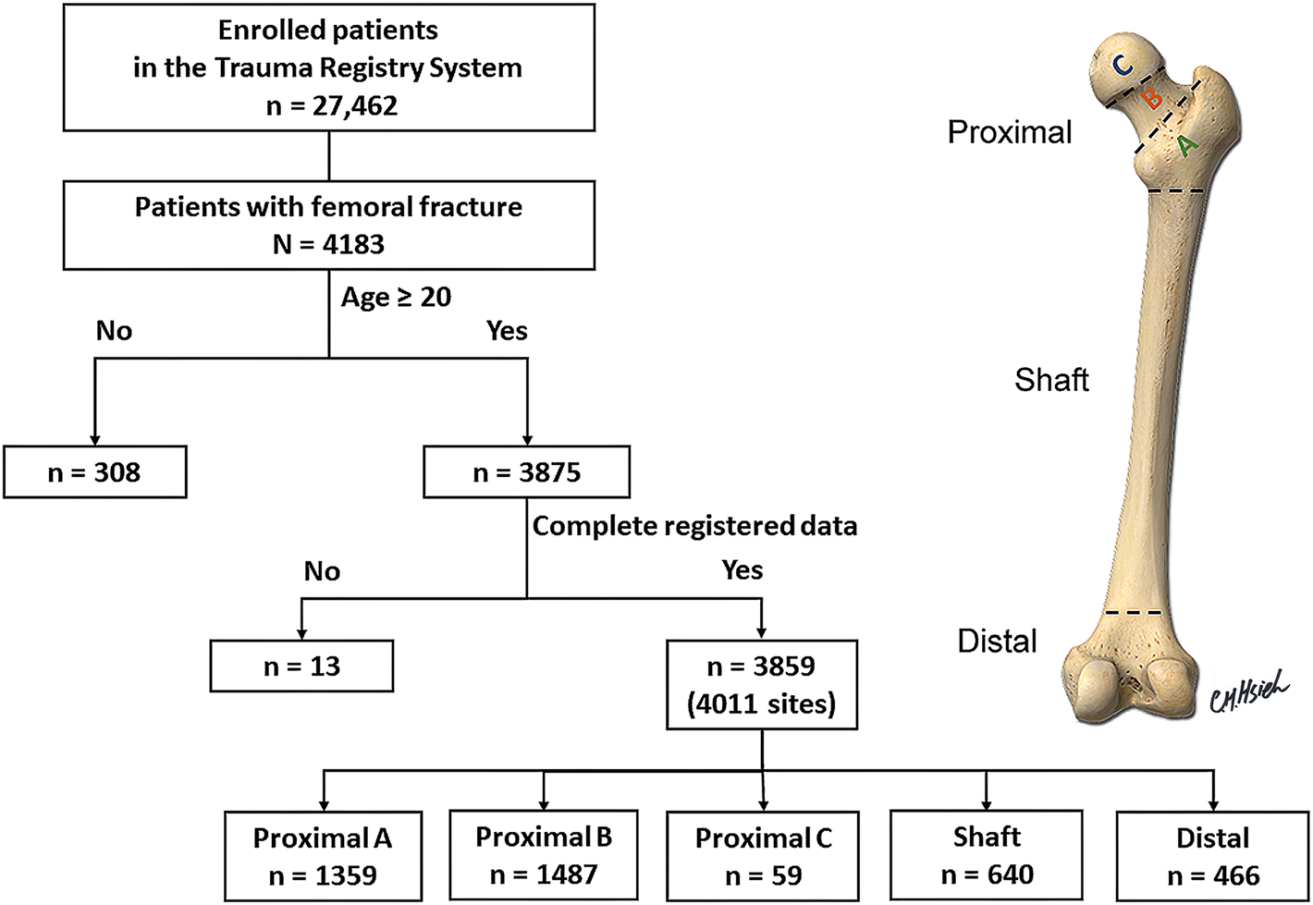
A flow chart presenting the grouping of fracture sites among the hospitalized adult patients with traumatic femoral bone fractures, based on the AO classification. There are five femoral fracture sites: proximal type A (trochanteric), proximal type B (neck), proximal type C (head), femoral shaft, and distal femur

This was a retrospective study and the work has been reported according to the guideline of Strengthening the Reporting of Cohort Studies in Surgery (STROCSS) [21]. This retrospective study reviewed data from 27,462 trauma patients registered between January 1, 2009 and December 31, 2016 (Fig. 1). The inclusion criteria required patients to be aged ≥20 years and hospitalized for the treatment of femoral fracture following injury. Patients with incomplete data were excluded. The patient data that was retrieved included age; sex; mechanism of trauma; BMI (which was calculated as weight (kg)/height (m)^2^). Patients were classified as obese (BMI of ≥30 kg/m^2^), overweight (BMI of < 30 but ≥25 kg/m^2^), normal weight (BMI of < 25 but ≥18.5 kg/m^2^), and underweight (BMI of < 18.5 kg/m^2^) based on the definition recommended by the World Health Organization [22, 23]. Trauma patients were categorized as faller (fall from standing height) or non-faller (which included motor vehicle accidents, motorcycle accidents, bicycle accidents, and patients struck by/against an object). According to the femoral fracture locations, 3859 patients with 4011 fracture sites were grouped into five subgroups as patients with fracture of proximal type A (*n* = 1359), proximal type B (*n* = 1487), proximal type C (*n* = 59), femoral shaft (*n* = 640), and distal femur (*n* = 466).

### Statistical analysis

Statistical analysis was performed using SPSS for Windows version 22 (IBM Corp., Armonk, NY, USA). For each subgroup of patients, the categorical variables of the patients were compared using a Pearson chi-squared test or Fishers exact test and presented as odds ratios (ORs) with 95% confidence intervals (CIs). The continuous variables were estimated using Levene’s test for homogeneity of variance and evaluated using one-way analysis of variance (ANOVA) with a Games–Howell post-hoc test to analyze the differences between the patients with or without femoral fracture at a specific site. Continuous data were expressed as mean ± standard deviation. Multivariate logistic regression analysis was applied to identify independent effects of univariate predictive variables on the occurrence of fracture at a specific site. A *p* value of < 0.05 was determined to be statistically significant. To provide an illustration of the pattern of patient characteristics in relation to the risk of occurrence of a femur fractures at a specific site, the propensity score which account for the risk of a fracture in a specific femoral site was calculated. This generated a scalar value defined as the conditional probability of assignment to an event, given a vector of observed covariates [24, 25]. These propensity scores and the factor of age were presented visually in a two-dimensional plot using the Generic X-Y Plotting functions in the pROC package in R (version 3.3.3.).

## Results

### The association of patient characteristics with the site of femoral fractures

As shown in the Tables 1 and 2, the patients who sustained proximal type A and B fractures were significantly older (78 vs. 69-years, *p* < 0.001 and 76 vs. 70-years, *p* < 0.001, respectively) than those that did not have femoral fracture in same location. In contrast, the patients who sustained proximal type C (35 vs. 73-years, *p* < 0.001), shaft (44 vs. 75-years, *p* < 0.001), and distal fractures (60 vs. 74-years, *p* < 0.001) were significantly younger than those who did not have a femoral fracture in same location. There were significantly fewer male patients with a proximal type B fracture (*p* < 0.001); however, there was a significant male predominance for proximal type C fracture (*p* < 0.001) and shaft fractures (*p* < 0.001). More patients who were involved in a fall had proximal type A fractures than those who did not have a fall (78.0% vs. 59.3%, *p* < 0.001). In contrast, fewer patients with proximal type B (22.0% vs. 57.0%, *p* < 0.001), C (16.9% vs. 66.6%, *p* < 0.001), distal (25.8% vs. 73.8%, *p* < 0.001) and shaft (37.8% vs. 69.7%, *p* < 0.001) fractures had been involved in a fall than those non-fallers. Patients who had proximal type A and B fractures had a significantly lower BMI (22.9 vs. 23.2, *p* < 0.001 and 22.5 vs. 23.6, *p* < 0.001, respectively) than those who did not have a femoral fracture in same location; in contrast, patients who had proximal type C (25.3 vs. 23.1, *p* < 0.001), shaft (24.3 vs. 22.9, *p* < 0.001) and distal fractures (25.0 vs. 22.9, *p* < 0.001) had a significantly higher BMI than those who did not have a femoral fracture in same location.

**Table 1:** The association of patient characteristics with proximal type A, B, and C femoral fractures

	Proximal A fracture			Proximal B fracture			Proximal C fracture		
	No	Yes	*p*	No	Yes	*p*	No	Yes	*p*
	*n* = 2500	*n* = 1359		*n* = 2372	*n* = 1487		*n* = 3800	*n* = 59	
Age (years)	69 [52, 79]	78 [67, 84]	< 0.001	70 [49, 81]	76 [66, 82]	< 0.001	73 [58, 81]	35 [26, 52]	< 0.001
Male, n (%)	1035 (41.4)	585 (43.0)	0.339	1095 (46.2)	525 (35.3)	< 0.001	1578 (41.5)	42 (71.2)	< 0.001
Fall, n (%)	1482 (59.3)	1060 (78.0)	< 0.001	1353 (57.0)	299 (22.0)	< 0.001	2532 (66.6)	10 (16.9)	< 0.001
BMI, median [IQR]	23.2 [20.8, 26.2]	22.9 [20.4, 25.6]	0.001	23.6 [21.0, 26.6]	22.5 [20.2, 25.0]	< 0.001	23.1 [20.7, 26.0]	25.3 [22.7, 28.7]	< 0.001
Weight, n (%)			0.001			< 0.001			0.029
Obese	327 (13.1)	148 (10.9)		332 (14.0)	143 (9.6)		464 (12.2)	11 (18.6)	
Overweight	611 (24.4)	309 (22.7)		624 (26.3)	296 (19.9)		900 (23.7)	20 (33.9)	
Normal weight	1367 (54.7)	746 (54.9)		1223 (51.6)	890 (59.9)		2086 (54.9)	27 (45.8)	
Underweight	195 (7.8)	156 (11.5)		193 (8.1)	158 (10.6)		350 (9.2)	1 (1.7)	

*BMI* Body mass index, *IQR* Interquartile range

**Table 2:** The association of patient characteristics with femoral shaft and distal femoral fractures

	Shaft fracture			Distal fracture		
	No	Yes	*p*	No	Yes	*p*
	*n* = 3219	*n* = 640		*n* = 3393	*n* = 466	
Age (years)	75 [63, 83]	44 [26, 65]	< 0.001	74 [60, 82]	60 [41, 73]	< 0.001
Male, n (%)	1256 (39.0)	364 (56.9)	< 0.001	1428 (42.1)	192 (41.2)	0.754
Fall, n (%)	2377 (73.8)	165 (25.8)	< 0.001	2366 (69.7)	176 (37.8)	< 0.001
BMI, medium [IQR]	22.9 [20.5, 25.8]	24.3 [21.6, 27.1]	< 0.001	22.9 [20.4, 25.7]	25.0 [22.2, 28.2]	< 0.001
Weight, n (%)			< 0.001			< 0.001
Obese	365 (11.3)	110 (17.2)		383 (11.3)	92 (19.7)	
Overweight	736 (22.9)	184 (28.7)		766 (22.6)	154 (33.0)	
Normal weight	1799 (55.9)	314 (49.1)		1904 (56.1)	209 (44.8)	
Underweight	319 (9.9)	32 (5.0)		340 (10.0)	11 (2.4)	

*BMI* Body mass index, *IQR* Interquartile range

### Independent variables of patient characteristics associated with sites of femoral fractures

As shown in Table 3, multivariate logistic regression analysis was applied to identify the independent variables that were associated with the occurrence of femoral fracture at a specific site. This analysis revealed that older age was an independent variable that could positively predict the occurrence of proximal type A (OR [95%CI]: 1.03 [1.03–1.04], *p* < 0.001) and B fractures (1.02 [1.01–1.02], *p* < 0.001), and negatively predict the occurrence of proximal type C (0.96 [0.94–0.98], *p* < 0.001), shaft (0.95 [0.95–0.96], *p* < 0.001), and distal fractures (0.98 [0.98–0.99], *p* < 0.001). In terms of proximal type A fractures, males had a 70% greater risk (OR = 1.70), when compared to females, and those who had a fall had a 47% greater risk (OR = 1.47), when compared to non-fallers. In terms of proximal type B fractures, fallers had a 93% greater risk (OR = 1.93), when compared to non-fallers, but those who were obese had a 28% lower risk (OR = 0.72) than those who were not obese. In terms of proximal type C and distal fractures, those who had a fall had a 63% lower risk (OR = 0.37) than those non-fallers. Regarding distal fracture, males and fallers had a 45 and 65% lower risk (OR = 0.55 and 0.35, respectively) than females and those non-fallers, respectively, but those obese patients had a 70% greater risk (OR = 1.70) than those who were not obese.

**Table 3:** Variables applied to multivariate logistic regression analysis to identify the independent factors associated with the occurrence of femoral fracture at a specific site

Fracture site	Proximal A			Proximal B			Proximal C		
Variables	coefficient	OR (95%CI)	*p*	coefficient	OR (95%CI)	*p*	coefficient	OR (95%CI)	*p*
Age	0.03	1.03 (1.03–1.04)	< 0.001	0.02	1.02 (1.01–1.02)	< 0.001	−0.04	0.96 (0.94–0.98)	< 0.001
Male	0.53	1.70 (1.46–1.98)	< 0.001	− 0.11	0.90 (0.78–1.04)	0.15	0.39	1.48 (0.83–2.77)	0.20
Faller	0.39	1.47 (1.22–1.77)	< 0.001	0.66	1.93 (1.61–2.31)	< 0.001	− 0.99	0.37 (0.16–0.83)	0.02
obese	−0.04	0.96 (0.77–1.19)	0.73	−0.33	0.72 (0.58–0.89)	< 0.001	0.24	1.27 (0.61–2.42)	0.50
Intercept	−3.33	0.04 (0.02–0.05)	< 0.001	−2.04	0.13 (0.09–0.18)	< 0.001	−1.78	0.17 (0.07–0.40)	< 0.001
Fracture site	Shaft			Distal					
Variables	coefficient	OR (95%CI)	*p*	coefficient	OR (95%CI)	*p*			
Age	−0.05	0.95 (0.95–0.96)	< 0.001	−0.02	0.98 (0.98–0.99)	< 0.001			
Male	−0.20	0.82 (0.66–1.01)	0.07	− 0.60	0.55 (0.44–0.69)	< 0.001			
Faller	−1.00	0.37 (0.29–0.47)	< 0.001	− 1.06	0.35 (0.27–0.45)	< 0.001			
obese	0.28	1.32 (1.00–1.74)	0.05	0.53	1.70 (1.30–2.20)	< 0.001			
Intercept	1.89	6.65 (4.62–9.62)	< 0.001	−0.18	0.84 (0.58–1.21)	0.35			

### An illustration of the pattern of patient characteristics in relation to the risk of occurrence of a fracture

**Fig. 2:**
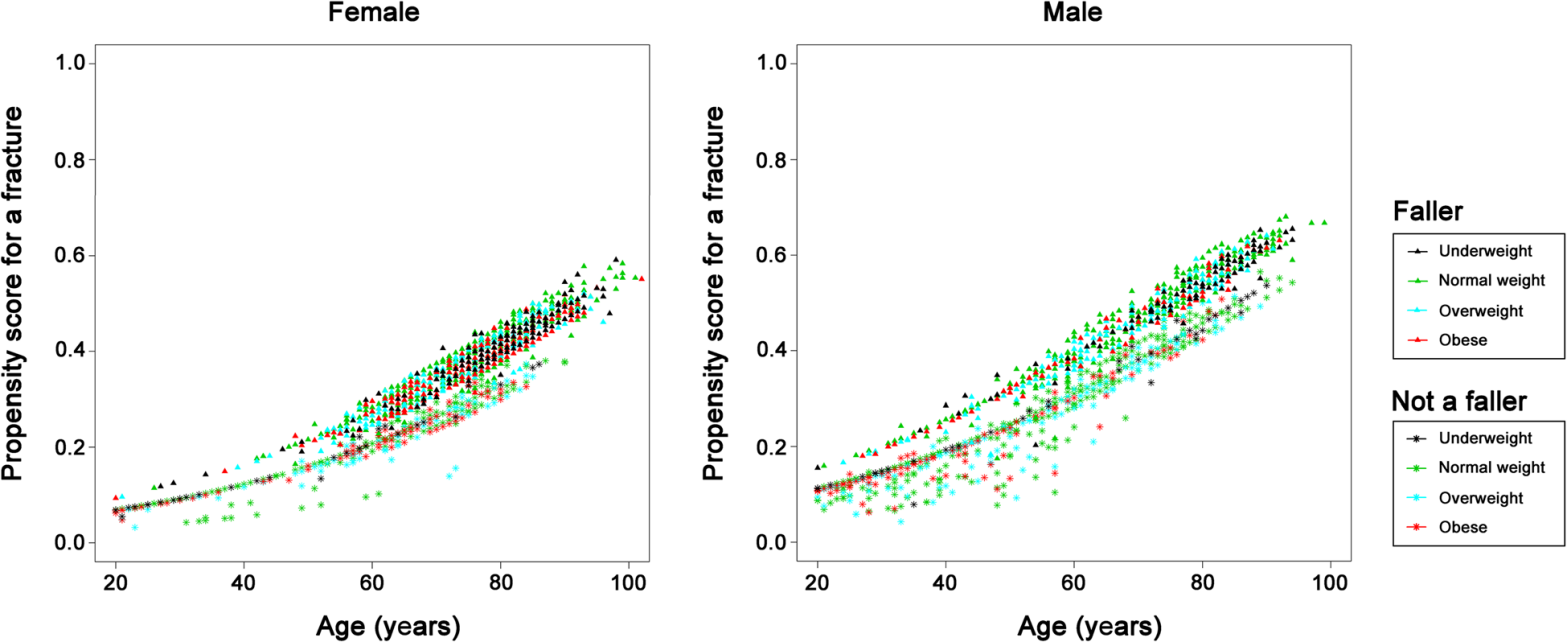
Plots showing fracture risk for proximal type A fractures in the female and male patients. X-axis: Age; Y-axis: Propensity score for the occurrence of a proximal type A fracture

**Fig. 3:**
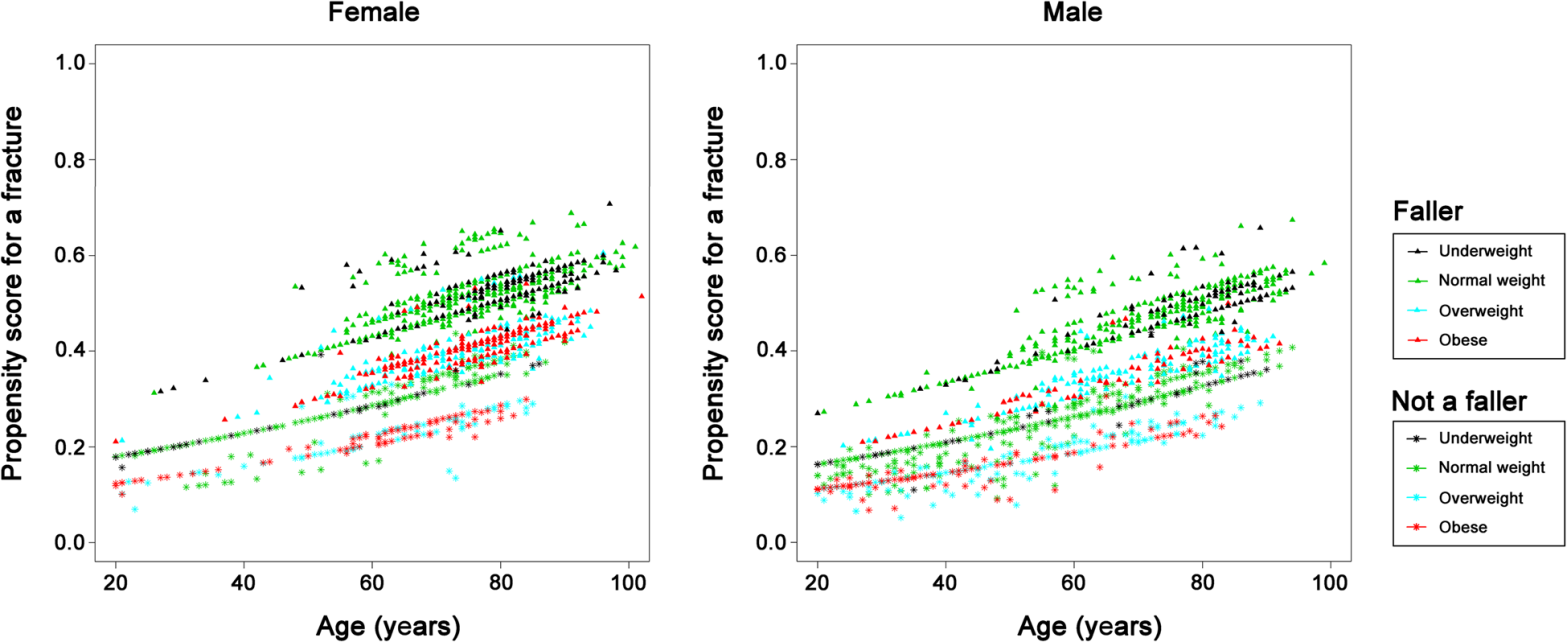
Plots showing fracture risk for proximal type B fractures in the female and male patients. X-axis: Age; Y-axis: Propensity score for the occurrence of a proximal type B fracture

The relationships between fracture risk patterns and patient characteristics in those who sustained proximal type A (Fig. 2) or B (Fig. 3) fractures are similar and revealed that, in these two fracture sites, older patients had a higher risk of fractures, the patients with a fall had a higher risk of sustaining a fracture, while those who were obese were at a lower risk of developing fractures in these sites. The incidence of proximal type A and B fractures increased with age both in the female and male patients.

**Fig. 4:**
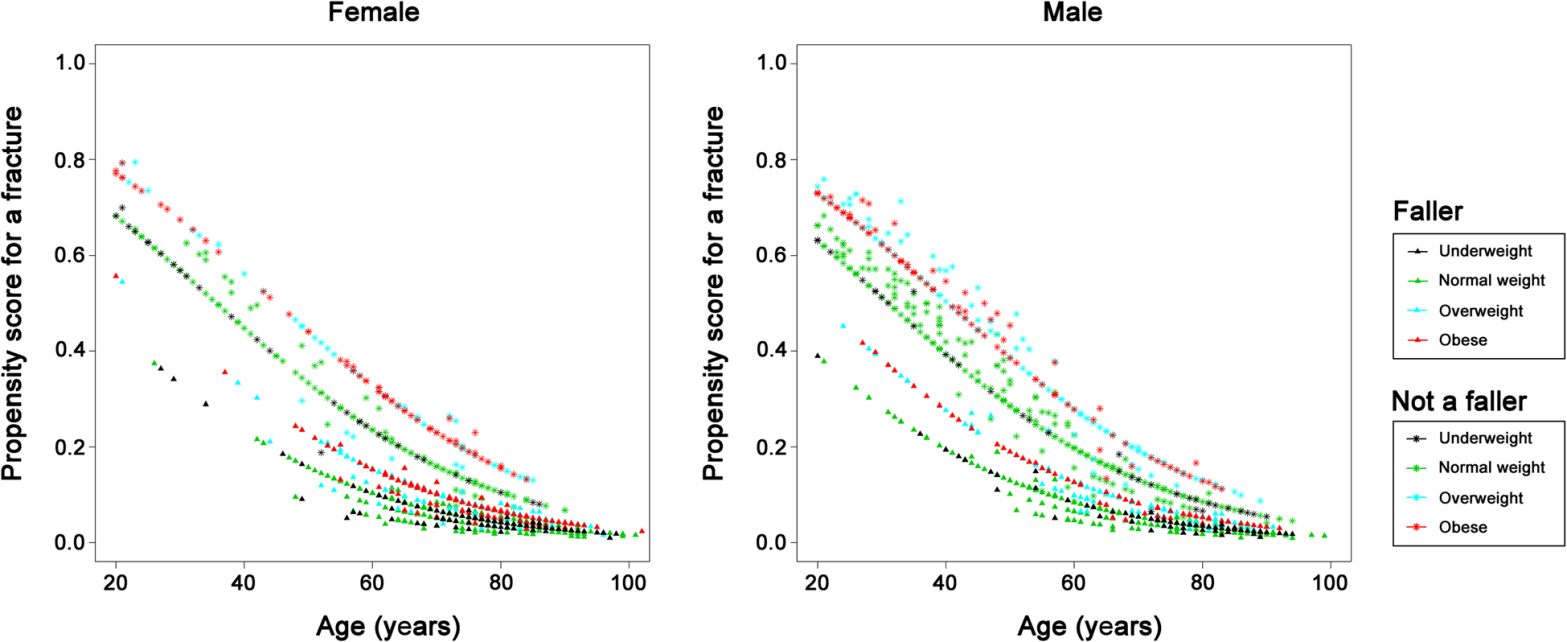
Plots showing fracture risk for femoral shaft fractures in the female and male patients. X-axis: Age; Y-axis: Propensity score for the occurrence of a femoral shaft fracture

**Fig. 5:**
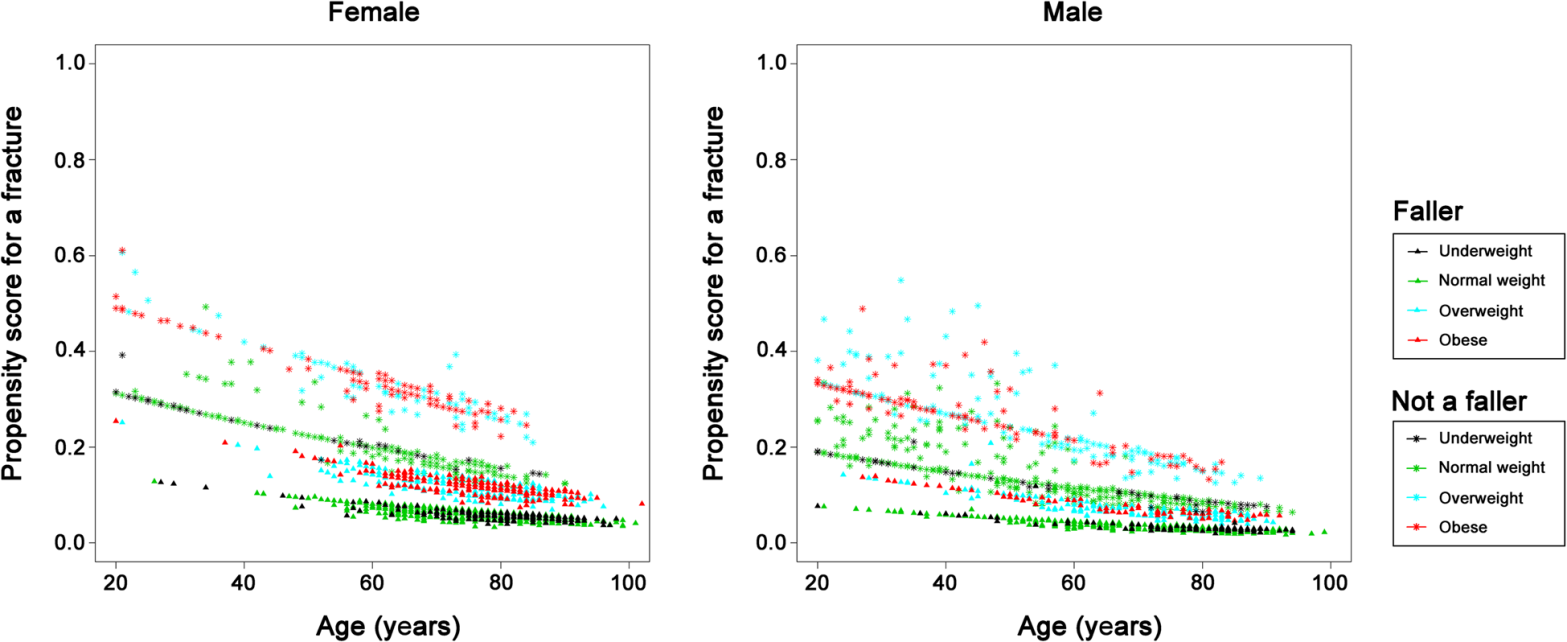
Plots showing fracture risk for fractures of the distal femur in the female and male patients. X-axis: Age; Y-axis: Propensity score for the occurrence of a fracture of the distal femur

**Fig. 6:**
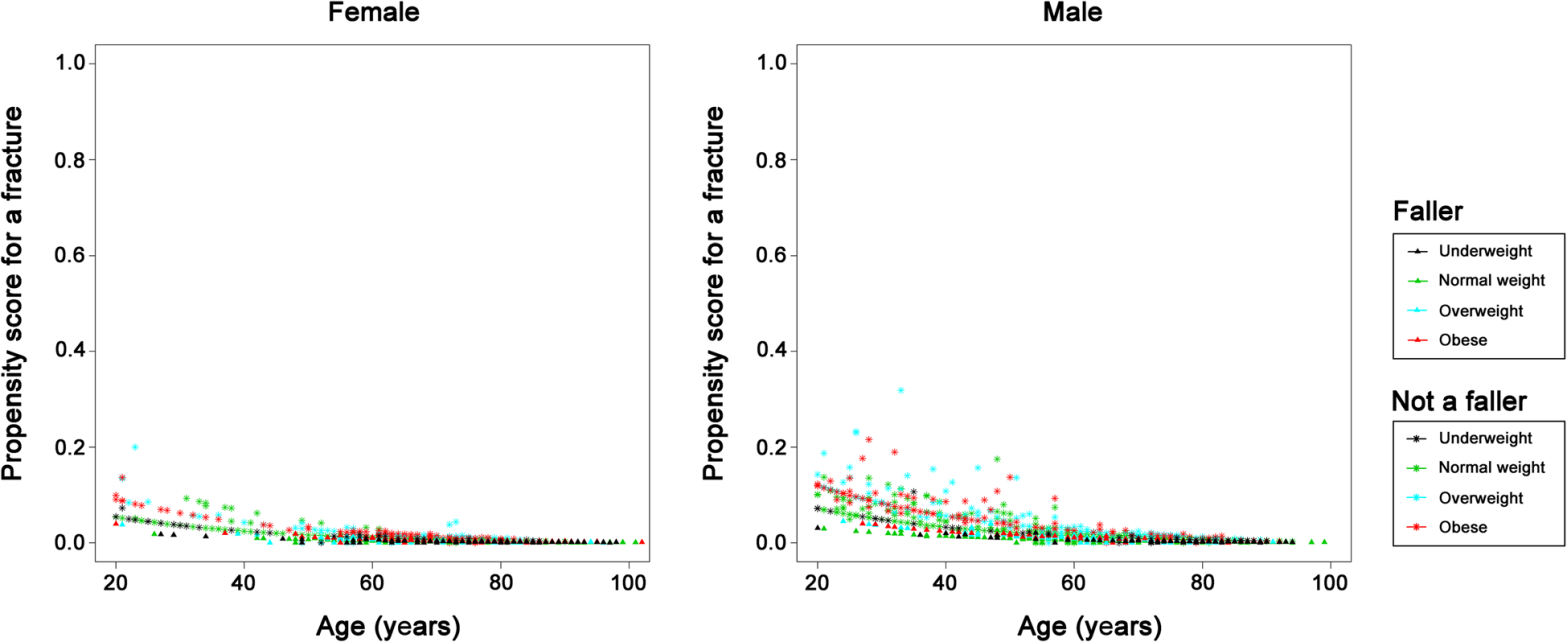
Plots showing fracture risk for proximal type C fractures in the female and male patients. X-axis: Age; Y-axis: Propensity score for the occurrence of a proximal type C fracture

The relationships between fracture risk patterns and patient characteristics in those who sustained a shaft (Fig. 4) or distal (Fig. 5) fracture are similar. The results are contrary to those observed for patients who had proximal type A or type B fractures. As noted in the illustrations, in the shaft and distal fracture sites, older patients and patients who had suffered a fall had a lower risk of sustaining these types of fracture, while those who were obese were at a higher risk of developing these fractures. The risk of developing shaft and distal femoral fractures decreased with age in both female and male patients. Furthermore, the fracture risk pattern for proximal type C fractures (Fig. 6) was similar to that observed in those who had a shaft or distal fracture, however, the trend is less prominent in this case and is less obvious because there were only a small number of patients with a proximal type C fractures (*n* = 59).

## Discussion

This study revealed a difference in the involvement of age in the incidence of femoral fracture sites in the trauma patients. Older patients were at a higher risk of developing proximal type A and type B fractures, while a lower risk of developing fractures in the shaft and distal femur. This incidence of fracture site can largely be explained by age-related factors, including a decrease in bone strength and falling being the most common mechanism of trauma in older patients. In cases where trauma is caused by a fall, the force directly impacts the posterolateral aspect of the greater trochanter, making the femoral neck particularly vulnerable to fractures [26]. Fall has been reported to be the major cause of intracapsular neck and peritrochanteric fractures of the femur [27], which are closely associated with severe generalized bone loss, and account for a significant proportion of femoral fractures in the elderly [28, 29]. In obese patients, increased soft tissue padding around the hip attenuates the greater impact forces that result from falls and reduces the force transmitted to the bone [15, 16]. In contrast, obese patients had a higher odds risk for shaft and distal femoral fractures in fall accidents [4]. In this type of scenario, the body weight associated with obesity would increase the impact force but provide less protection in the shaft and distal region of the femur.

Femoral shaft and distal fractures can occur in younger patients without any evidence of osteoporosis, usually as a result of a high-energy trauma such as a motor vehicle accident or motorcycle accident [8, 9, 30, 31]. The impact of the energy transmitted to the bone is generally greater in traffic accidents than in fall accidents, and the point of impact to the femur is not limited to the greater trochanter, which commonly occurs in falls. In cases involving high-energy trauma to various sites of the femur, obesity does not reduce the impact, and may even increase the trauma by applying an unbearable load to the bone, as the magnitude of impact force increases in proportion to body weight. For example, it has been demonstrated that in motor vehicle accidents, an elevated BMI decreases the risks of injury in nearside impacts, but increases the risk of lower-extremity injury in frontal crashes [15, 16].

Increasing age has been reported to be associated with an increase in the incidence of proximal type A fractures in women and an increase in the incidence of proximal type B and C fractures in men [32]. In addition, previous studies have demonstrated that the proportion of trochanteric fractures increased with age in female patients but not in male patients [30, 33, 34]. This phenomenon is believed to be due to more pronounced bone loss in older female patients compared to males, which makes female subjects more susceptible to peritrochanteric fractures than intracapsular fractures [30, 33, 34]. In this study, we did not find a difference between genders, and, as patients increased in age, the trend for both female and male patients to sustain a fracture in a given femoral site was similar. While the incidence of proximal type A and B fractures increased with age in both genders, the incidence of shaft and distal femoral fractures decreased with age in both genders. Female gender is independently associated with a higher rate of a proximal type A fractures and a lower rate of distal fractures.

In this study, the head of the femur was the part that was the least likely to be fractured, probably because it is relatively hidden in the acetabulum, therefore would only fracture if an impact force was transmitted from the femur to the hemi pelvis and acetabulum. Although the fracture risk pattern in proximal type C fractures was similar to that observed in those who had a shaft or distal fracture, the trend is less clear due to the small number of patients with this type of fracture. This makes it harder to draw conclusions about fractures at this site.

This study has some limitations that need to be acknowledged. First, the retrospective design of the study may have resulted in a selection bias. Second, the impact force, impact location and the protection method were unknown during the trauma accident, especially considering these factors may vary widely during a high-speed motorcycle accidents. Third, the lack of information regarding the status of osteoporosis may lead to a bias in the analysis. Fourth, this study does not cover a number of risk factors, which have been identified for as risk factors for fracture including medication use such as glucocorticoids [35, 36] or bisphosphonates [37], previous fragility fractures [38], and smoking and alcohol consumption [39], which may result in a bias in the analysis. At last, the population included in this study is limited to a single urban trauma center in southern Taiwan, which means that the results may be different in other regions.

## Conclusion

This study revealed that the fracture risk for developing proximal type A and B fractures increases with aging in a similar pattern, which is in contrast to the fracture risk pattern for shaft or distal fractures.

## Data Availability

The data are not publicly available due the privacy of patients included but are available from the corresponding author on reasonable request for academic research purpose.

## Abbreviations

ANOVA: One-way analysis of variance
AO: Arbeitsgemeinschaft für Osteosynthesefragen
BMD: bone mineral density
CI: Confidence intervals
IRB: Institutional review board
OR: Odds ratio
STROCSS: Strengthening the Reporting of Cohort Studies in Surgery
